# Patellar Tendon Stiffness Is Not Reduced During Pregnancy

**DOI:** 10.3389/fphys.2019.00334

**Published:** 2019-03-29

**Authors:** Marie Elena Bey, Robert Marzilger, Larry Hinkson, Adamantios Arampatzis, Kirsten Legerlotz

**Affiliations:** ^1^Department of Training and Movement Sciences, Humboldt-Universität zu Berlin, Berlin, Germany; ^2^Berlin School of Movement Science, Berlin, Germany; ^3^Department of Obstetrics, Charité – Universitätsmedizin Berlin, Berlin, Germany

**Keywords:** tendon, stiffness, length, muscle strength, exercise, laxity, injury, pregnancy

## Abstract

It is believed that hormonal changes during pregnancy lead to an increased compliance in ligaments and tendons, increasing the risk to suffer from connective tissue injuries particularly during exercise. While the laxity of the pelvic ligaments may increase to facilitate childbirth, to our knowledge no study has ever investigated the mechanical properties of human tendons in different stages of pregnancy. Thus, the purpose of our longitudinal study was to investigate the mechanical properties of the patellar tendon in different stages of pregnancy and postpartum. Nineteen pregnant women (30 ± 4 years) and 11 non-pregnant controls (28 ± 3 years) performed maximum isometric knee extension contractions on a dynamometer. Muscle strength and mechanical properties of the patellar tendon were determined integrating ultrasound, kinematic, and electromyographic measurements. In pregnant women, measurements were performed in the 16 ± 4th week of pregnancy (EP), the 29 ± 4th week of pregnancy (LP) and 32 ± 9th weeks postpartum (PP). On average, muscle strength as well as patellar tendon stiffness, force, and relative strain did not change during pregnancy and did not differ from non-pregnant controls. Tendon length measured at 90° knee flexion continuously increased during and after pregnancy (tendon length PP>EP; PP>controls). Our results indicate that patellar tendon stiffness is not universally affected by pregnancy. We found no evidence to support the often stated assumption that tendons would become more compliant during pregnancy. However, variability between individuals as well as the progressive increase in tendon rest length during and after pregnancy and its implications on injury risk need to be further examined.

## Introduction

It is well established that physical activity during pregnancy has beneficial effects on maternal and fetal health (Vladutiu et al., 2010) decreasing the risk of pregnancy associated disorders such as preeclampsia or gestational diabetes (DeMaio and Magann, 2009). Whilst pregnant women are encouraged to pursue low-impact activities such as aerobic training or walking to maintain their cardiovascular fitness (DeMaio and Magann, 2009; Nascimento et al., 2012), it has been recommended to avoid high-intensity exercise (DeMaio and Magann, 2009). During excessive physical activity pregnant women are thought to be prone to overheating (Sasaki et al., 1995), since their core temperature has been reported to increase with the growing fetus (Buxton and Atkinson, 1948). In addition, strenuous exercise may reduce blood flow to the placenta, which may impair fetal development (Rauramo and Forss, 1988). In order to maintain overall body strength and to improve body posture or reduce back pain during pregnancy (Zavorsky and Longo, 2011) strength training with low weights and low intensity has been suggested.

However, even when moderate exercise is undertaken, pregnant women are often cautioned that hormonal changes during pregnancy may increase ligament and tendon compliance (Östgaard et al., 1993; Dumas and Reid, 1997; Ritchie, 2003) possibly leading to connective tissue injuries and joint pain, such as patellofemoral dysfunction (Ritchie, 2003; Harland et al., 2014). Increased connective tissue compliance is further believed to cause joint instability (Ritchie, 2003) which may impair postural stability. Indeed, recent studies demonstrated an increase in postural sway (Jang et al., 2008; Oliveira et al., 2009) as well as impairments in dynamic (Inanir et al., 2014) and static postural stability, already occurring in the early stages of pregnancy (Bey et al., 2018). Since impairments in postural stability are associated with the high incidence of falls in pregnant women (Dunning et al., 2010) this may, in turn, lead to further injuries.

It has been known for decades and has been well documented that the compliance of the pubic ligaments increases during pregnancy which is an essential process to facilitate childbirth (Young, 1940). Recent studies suggest that the laxity of the peripheral joints might similarly increase during pregnancy, with a greater range of motion in the knee joint (Schauberger et al., 1996), the elbow (Schauberger et al., 1996), the wrist (Marnach et al., 2003), and the metacarpophalangeal joints (Calguneri et al., 1982; Schauberger et al., 1996) being reported. While those studies in humans were drawing conclusions on connective tissue properties from range of motion changes, not directly measuring tissue properties, one study in pregnant rabbits has actually determined the stiffness of the medial collateral ligament by *in vitro* material testing (Hart et al., 2000). This study did not find any effect of pregnancy on structural, material and viscoelastic properties of the rabbit’s medial collateral ligament.

To our knowledge, in pregnant women the mechanical properties of tendons and ligaments in peripheral joints have never been investigated. However, hormonal changes occurring during pregnancy may affect connective tissue properties even in peripheral regions of the human body, thereby possibly increasing the risk of injury. Hormonal fluctuations during the menstrual cycle such as increased levels of relaxin (hRLX) have already been reported to be associated with a decreased patellar tendon stiffness (Pearson et al., 2011). As hRLX levels are also elevated in pregnant women, being ten times larger compared to levels occurring in non-pregnant women (MacLennan et al., 1986), the hormonal effect on the patellar tendon is expected to be potentiated during pregnancy. However, hormonal effects on tendons are likely to be tendon specific since levels of hRLX during the menstrual cycle were not related to the gastrocnemius tendon stiffness (Pearson et al., 2011).

The aim of our longitudinal study was to investigate the mechanical properties of the patellar tendon at two different stages of pregnancy and 6 months after delivery. In addition, the postpartum values were compared to non-pregnant controls. We hypothesized that patellar tendon stiffness decreases during pregnancy. Quantifying the effect of pregnancy on the mechanical properties of tendons may help to better assess injury risk and to derive from that appropriate recommendations regarding physical activity during pregnancy. Further, our study may contribute to a better understanding of hormonal effects on female connective tissue properties in general, as also in other contexts hormonal variations are discussed to affect injury risk (e.g., association of anterior cruciate ligament rupture incidence with certain phases of the menstrual cycle) (Herzberg et al., 2017).

## Materials and Methods

### Participants

Twenty-one pregnant and eleven non-pregnant healthy non-sedentary women agreed to participate in this study. Two pregnant women dropped out of the study due to medical issues in the late stage of pregnancy. Therefore, 19 pregnant and 11 non-pregnant controls completed the study. Previous studies have shown that this sample size is sufficient to detect hormone related or training induced changes in patellar tendon stiffness (Pearson et al., 2011; Mersmann et al., 2016). In 12 menstruating women, Pearson et al. (2011) found a significant correlation (*r* = -0.560, *p* < 0.001) between hRLX hormone levels and patellar tendon stiffness. Another study with 12 participants detected a 4% increase in patellar tendon stiffness (*p* = 0.003) after a 12 months of sport-specific training (Mersmann et al., 2016).

In the pregnant women patellar tendon properties and the maximum knee joint moment were determined in the early stage of pregnancy [EP, 16 ± 4 week of pregnancy (WoP)], the late stage of pregnancy (LP, 29 ± 4 WoP) and at least 6 months postpartum (PP, 32 ± 9 weeks). Except for two participants (Table 3) a measurement prior to pregnancy was not possible. Thus, the time-point for the postpartum measurement was chosen to reflect the non-pregnant status assuming that 6 months after delivery the women would have recovered from childbirth and hormone levels would have returned to pre-pregnancy levels. A study by Schauberger et al. (1996) has demonstrated that increased levels of hRLX during pregnancy returned to pre-pregnancy levels within 2 weeks postpartum.

In the non-pregnant controls the same variables were determined once. Women with a multiple pregnancy, severe pathological pregnancy associated symptoms and present or past injuries of the knee were excluded from the study. Pregnant women (30 ± 4 years) were on average 2 years older than the non-pregnant controls (28 ± 3 years). This study was carried out in accordance with the recommendations of the local ethics committee Charité – Universitätsmedizin Berlin with written informed consent from all subjects. All subjects gave written informed consent in accordance with the Declaration of Helsinki. The protocol was approved by the ethics committee Charité – Universitätsmedizin Berlin.

### Experimental Setup

**FIGURE 1:**
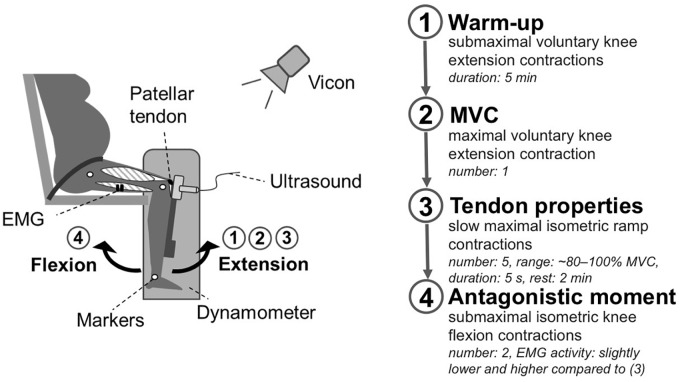
Schematic representation of the experimental setup. Participants were seated on a dynamometer with a 90° knee joint angle. In this position, the participants performed a 5 min warm-up with submaximal voluntary knee extension contractions (1). Thereafter, the women performed a maximal voluntary knee extension contraction to estimate the maximal voluntary moment (2). To determine tendon properties (3) the knee joint moment was assessed during five slow maximal isometric ramp contractions (∼80–100% MVC), considering EMG activity of the antagonists and movements of the knee joint relative to the dynamometer captured by Vicon. The tendon elongation was recorded using ultrasound. Subsequently, two submaximal isometric knee flexion contractions (4) were performed to estimate the antagonistic contribution to the measured knee joint moment during the ramp contractions.

Briefly, the women performed five slow maximal isometric ramp contractions and two submaximal isometric knee flexion contractions on a dynamometer. Length changes of the patellar tendon were recorded with ultrasound. Muscle strength of the knee extensors was assessed by the knee joint moment measured by the dynamometer. To take gravitational forces and a misalignment of the knee joint and the dynamometer axis during the contractions into account (Arampatzis et al., 2004) a motion capture system was used. To subtract the contribution of the antagonistic moment from the measured knee joint moment the antagonist muscle activity was measured using electromyographic (EMG) measurements (Mademli et al., 2004). All measurements were conducted with the dominant leg, defined as the commonly used leg for kicking a ball. For a schematic representation of the experimental setup see Figure 1.

#### Measurement of Maximum Knee Joint Moment

Participants were seated on a dynamometer (Biodex Medical System 3, Shirley, NY, United States) with a 90° resting knee joint angle (Pearson et al., 2011; Hansen et al., 2013) and an 85° trunk angle. To prevent hip movements the participants were fastened to the seat using a non-elastic strap. After a 5 min warm-up phase with submaximal voluntary knee extension contractions the women performed one maximal voluntary knee extension contraction (MVC) as well as five slow maximal isometric ramp contractions (∼80–100% MVC) with a steadily increasing effort to the maximum within 5 s, and 2 min rest between contractions.

To take gravitational forces and a misalignment of the knee joint and the dynamometer axis during contraction into account (Arampatzis et al., 2004), kinematic data were collected using a Vicon motion capture system (version 1.7.1; Vicon Motion Systems, Oxford, United Kingdom) integrating seven cameras at a frame rate of 250 Hz. Five reflective markers were captured which were positioned at the trochanter major, lateral, and medial epicondyle of the femur as well as the lateral and medial malleolus.

To determine the resultant knee extensor moment without the antagonistic contribution of the knee flexors during the ramp contractions, the antagonistic moment was subtracted from the measured knee joint moment. The antagonistic moment was estimated by establishing the relationship of the EMG activity of the knee flexors during the ramp contractions and the exerted moment of the knee flexors during knee flexion contractions, when acting as agonists (Mademli et al., 2004). The EMG activity of the flexors was recorded with one pair of bipolar surface electrodes (Myon m320RX; Myon, Baar, Switzerland) which were placed centrally over the long head of the biceps femoris in the direction of the muscle fibers. The sample rate was set at 1000 Hz. The exerted moment was measured during two submaximal isometric knee flexion contractions with an intensity resulting in a slightly lower and higher activity than the previously determined activity during the ramp contractions (Mademli et al., 2004).

#### Measurement of Mechanical Tendon Properties

Patellar tendon elongation during the knee extension contractions was analyzed in the sagittal plane using a 10 cm ultrasound probe (7.5 MHz, My Lab60, Esaote, Genova, Italy). Ultrasound images were captured at 25 Hz. Externally induced trigger signals set in the beginning and the end of the ramp contractions facilitated the synchronization of the ultrasound images and the kinematic data.

**FIGURE 2:**
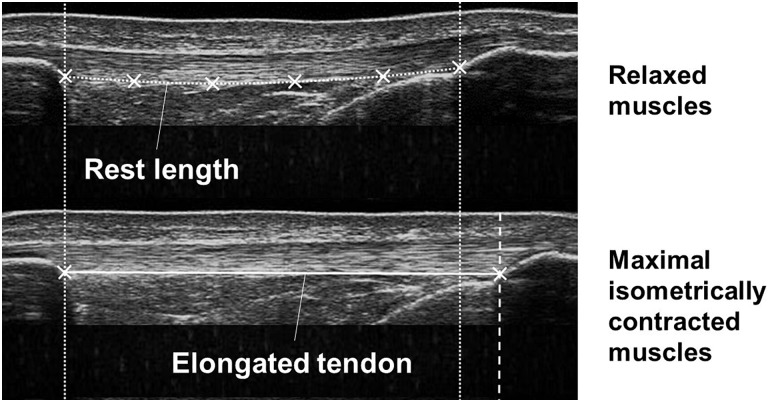
Ultrasound images demonstrating the tendon in the relaxed (upper image) and maximal isometrically contracted state (lower image) of the muscles. Tendon rest length and tendon elongation were measured defining reference points at the patellar apex and the tibial tuberosity and the deep boundary of the tendon.

Using a custom written Matlab interface (version R2012a; MathWorks, Natick, MA, United States), the patellar tendon elongation was analyzed frame by frame manually tracking the deep insertion of the tendon at the patellar apex and the tibial tuberosity (Mersmann et al., 2014). Tendon rest length at 90° knee joint angle was defined as tendon length in the inactive state of the muscles being determined by tracing the deep boundary of the tendon (Figure 2). In a 90° knee flexion position the patellar tendon may have been subjected to a small pretension, thus tendon length in a 90° knee flexion position may differ from the true rest length. To examine the variation of the rest lengths within each stage of pregnancy we calculated the standard deviation of the rest lengths for each participant separately and determined from the results the overall mean and standard deviation. The within-day variation was 0.77 ± 0.66 mm for EP, 0.71 ± 0.51 mm for LP, and 0.77 ± 0.38 mm for PP. Tendon elongation was measured in the active state of the muscles when the rest length was exceeded.

**FIGURE 3:**
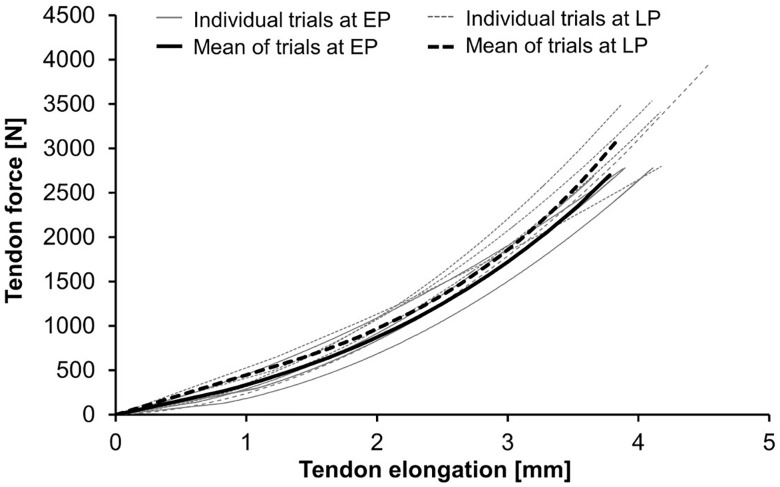
Tendon force-elongation ratios at the early (EP) and late (LP) stage of pregnancy. Demonstrated are the individual trials and their means in one pregnant woman (for data see Woman-A in Table 3).

To determine the tendon relative strain, the maximum elongation was normalized to the tendon rest length. Tendon force was calculated dividing the previously measured knee extension moment by the tendon moment arm, which was predicted based on the body height and the body mass from the PP measurement (Mersmann et al., 2016). After calculating the average of five tendon force-elongation ratios (Schulze et al., 2012), the resultant force-elongation curve was fitted using a second-order polynomial. Examples for force-elongation ratios during pregnancy are presented in Figure 3. Tendon stiffness was defined as the slope of a regression line between 50% and 100% of the maximum tendon force. Toe limit elongation was obtained as abscissa of the intersection point of the regression line and the zero force axis (Seynnes et al., 2013).

### Statistical Analysis

Statistical analysis was performed using IBM SPSS Statistics (Version 21, 32 Bit, IBM, United States). Normality of the standardized residuals was analyzed conducting the Shapiro-Wilk test. Differences between EP, LP, and PP were investigated using a one-way repeated measures ANOVA considering the assumption of sphericity. If the assumption of sphericity was violated the Greenhouse–Geisser correction was used. For *post hoc* comparisons paired *t*-tests with Bonferroni adjustment were performed. If the data were not normally distributed the Friedman’s test and the Wilcoxon signed-rank test for pairwise comparisons were conducted.

To compare the anthropometric measures (body height, moment arm, and age) and the postpartum session of the pregnant women with the non-pregnant controls either an independent samples *t*-test or the Mann–Whitney *U*-test for not normally distributed data were used. The effect size was calculated using Cohen’s d for normally distributed data. For not normally distributed data the effect size r was determined dividing the z-scores of the Wilcoxon or Mann–Whitney *U*-test by the square root of the number of total observations. Subsequently, the result r was converted into d. The alpha level for all statistical tests was set at α = 0.05.

## Results

### Anthropometric Measures

Body mass and body mass index (BMI) at LP were significantly higher compared to EP (d_mass_ = 3.29, *p* < 0.001; d_BMI_ = 3.65, *p* < 0.001) and PP (d_mass_ = 2.88, *p* < 0.001; d_BMI_ = 2.88, *p* < 0.001) (Table 1). Body mass and BMI in PP did not differ from non-pregnant controls. Body height (Table 1) and moment arm (Table 2) were significantly larger in the pregnant women compared to the non-pregnant controls (d_height_ = 0.99, *p* = 0.014; d_momentarm_ = 1.03, *p* = 0.011).

**Table 1:** Anthropometric data for the pregnant women in the early (EP) and late (LP) stage of pregnancy, postpartum (PP), and for the non-pregnant controls (means ± standard deviation).

		Body mass [kg]	Body height [cm]	Body mass index [kg/m^2^]
Groups	Week			
Controls	–	60.3 ± 5.5	165 ± 4	22.3 ± 2.2
EP	16 ± 4 WoP	66.2 ± 7.8	170 ± 6^#^	23.0 ± 2.9
LP	29 ± 4 WoP	72.3 ± 8.4*	–	25.1 ± 3.3*
PP	32 ± 9 after delivery	65.2 ± 10.8	–	22.6 ± 4.0


*^∗^significantly different to EP and PP (*p* < 0.05). ^#^significantly different to the controls (*p* < 0.05).*

**Table 2:** Knee extensor moment and patellar tendon properties for the pregnant women in the early (EP) and late (LP) stage of pregnancy, postpartum (PP), and for the non-pregnant controls (means ± standard deviation).

	Knee extensor moment [Nm]	Moment arm [mm]	Toe limit elongation [mm]	Maximum elongation [mm]
Groups				
Controls	144.5 ± 34.1	49.4 ± 0.7	0.92 ± 0.38	3.34 ± 0.73
EP	144.0 ± 34.8	–	1.00 ± 0.39	3.54 ± 0.70
LP	146.9 ± 37.1	–	1.02 ± 0.57	3.61 ± 0.82
PP	140.6 ± 33.9	50.5 ± 1.2^#^	1.29 ± 0.57°	3.79 ± 0.86


*^#^significantly different to the controls (*p* < 0.05). ^°^tendency toward a difference from the controls (*p* = 0.0501)*.

### Patellar Tendon Properties

**FIGURE 4:**
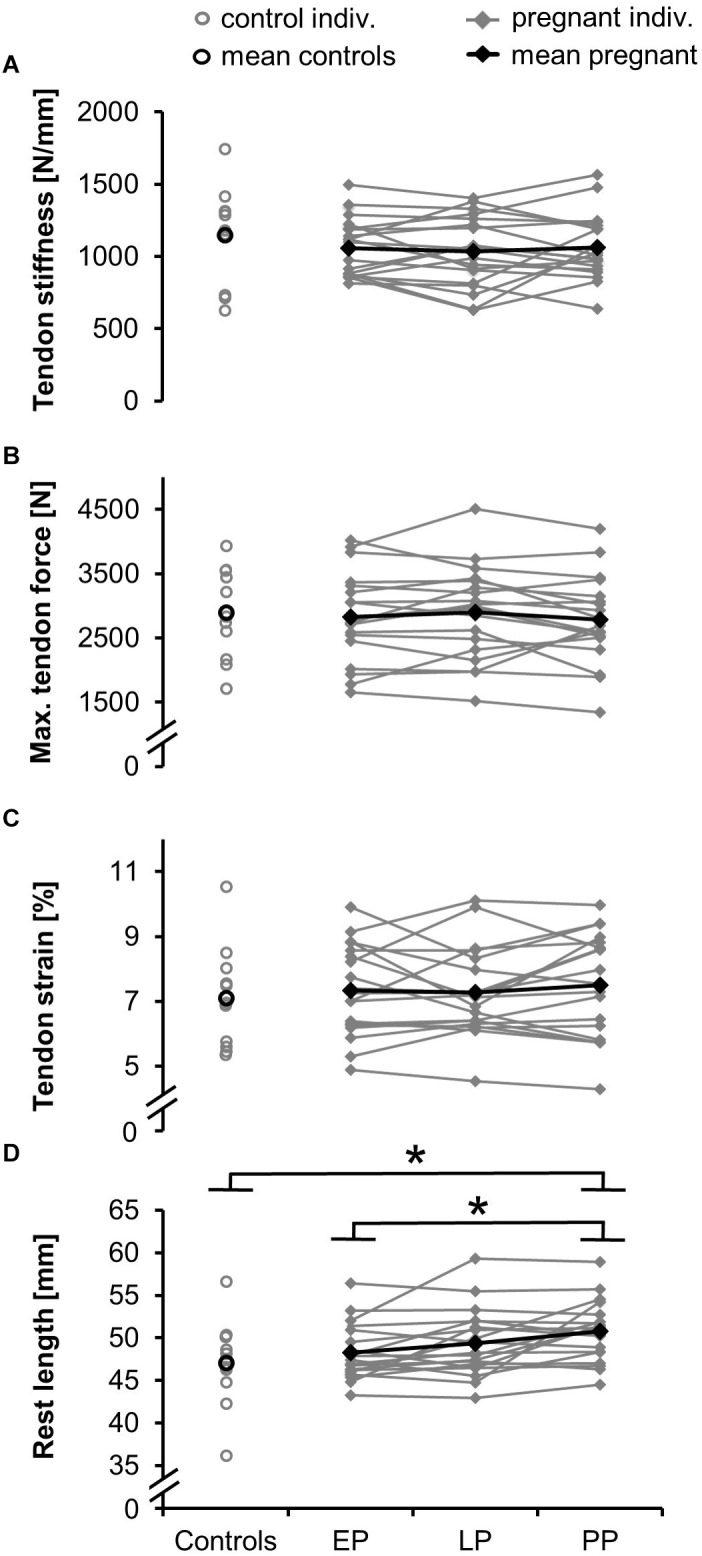
Means and individual data for the tendon stiffness **(A)**, maximum tendon force **(B)**, relative strain **(C)**, and rest length **(D)** in the pregnant women in the early (EP) and late (LP) stage of pregnancy, postpartum (PP) and in the non-pregnant controls (^∗^significantly different, *p* < 0.05).

Tendon stiffness (EP: 1,060 ± 195 N/mm, LP: 1,033 ± 238 N/mm, PP: 1,064 ± 220 N/mm) (Figure 4A) did not change during and after pregnancy. Similarly, for the knee extensor moment (Table 2), tendon relative strain (EP: 7.3 ± 1.4%, LP: 7.3 ± 1.3%, PP: 7.5 ± 1.6%) and maximum tendon force (EP: 2,832 ± 674 N, LP: 2,899 ± 700 N, PP: 2,781 ± 661 N) (Figure 4B,C) no significant differences were detected. Tendon rest length (Figure 4D) increased during and after pregnancy (EP: 48.2 ± 3.3 mm, LP: 49.3 ± 3.8 mm, PP: 50.6 ± 3.4 mm) being significantly larger in PP compared to EP (*d* = 0.732, *p* = 0.002). Maximum elongation and toe limit elongation (Table 2) did not change during and after pregnancy.

Postpartum rest length (Figure 4D) significantly increased compared to the non-pregnant controls (rest length = 47.1 ± 4.9 mm; *d* = 0.88, *p* = 0.028). For the toe limit elongation (Table 2) a tendency toward a difference from the controls (*d* = 0.71, *p* = 0.0501) was detected. The postpartum tendon stiffness (stiffness = 1,147 ± 321 N/mm), knee extensor moment, tendon moment arm, and maximum elongation (Table 2) were not significantly different between pregnant women and controls.

### Individual Data Sets

In two women we were able to obtain data at 37 and 36 weeks prior pregnancy in addition to the EP, LP, and PP time-points (Table 3). While it is not possible to statistically analyze those individual two data sets, the data are in agreement with the comparison of PP with non-pregnant controls. Both women show no reduction in tendon stiffness during pregnancy. While the tendon stiffness in Woman-A increased during pregnancy up to +17%, marginal changes up to +4% were found in Woman-B. In both women, rest length at a 90° knee joint angle was observed to increase during and after pregnancy (Woman-A: +16%, Woman-B: +6%).

**Table 3:** Changes in the body mass, knee extensor moment and patellar tendon properties for two women (Women-A: age 34 years, height 169 cm; Women-B: age 26 years, height 162 cm), who have been measured prior pregnancy, in the early (EP) and late (LP) stage of pregnancy as well as postpartum (PP).

Session	Week	Body mass [kg]	Knee extensor moment [Nm]	Rest length [mm]	Toe limit elongation [mm]	Maximum elongation [mm]	Relative Strain [%]	Tendon force [N]	Stiffness [N/mm]
**Woman-A**									
prior	37 pre	56.2	144.7	46.3	0.93	3.22	6.96	2,842	1,183
EP	12 WoP	55.5	159.2	53.2	1.39	3.72	7.00	2,729	1,117
LP	28 WoP	63.6	162.0	53.6	1.59	3.84	7.19	3,282	1,382
PP	31 post	57.4	156.8	52.8	1.93	4.53	8.59	3,150	1,193
**Woman-B**									
prior	36 pre	54.8	175.5	48.7	1.11	3.68	7.56	3,573	1,312
EP	14 WoP	57.7	162.3	48.6	0.56	2.85	5.87	3,365	1,358
LP	27 WoP	61.0	164.5	46.3	0.45	2.93	6.31	3,391	1,332
PP	25 post	52.4	147.1	51.5	0.92	3.32	6.45	3,026	1,216


## Discussion

This is the first study providing evidence on the mechanical properties of human tendons in different stages of pregnancy. Our study did not detect changes in patellar tendon mechanical properties during pregnancy. Therefore, we reject our hypothesis and conclude that tendon stiffness does not universally decrease during pregnancy.

While studies determining the mechanical properties of ligaments or tendons in pregnant mammals are rare, so far no animal study (Rundgren, 1974; Hart et al., 2000) has detected a reduced stiffness in tendons or ligaments of peripheral joints with pregnancy, confirming our results. A study in pregnant rabbits determined the structural, material, and viscoelastic properties of the medial collateral ligament by *in vitro* material testing and found no effect of pregnancy on ligament stiffness (Hart et al., 2000). Similar research in pregnant rats demonstrated that the mechanical properties of the posterior cruciate ligament were in general not affected by pregnancy, with no changes in stiffness during gestation being detected. Only for the first 3 days of the postpartum period a reduction in maximum load was recorded, returning to or above control levels thereafter (Rundgren, 1974). To our knowledge, this was also the only study that has ever investigated the mechanical properties of tendons during pregnancy, with no reduction in tendon stiffness of the rat musculus digiti quinti tendon being detected by material testing, neither during gestation nor in the postpartum period. In our study, the average tendon stiffness remained constant during and after pregnancy. Thus, there is no evidence warranting the statement that changes in tendon tissue mechanical properties would increase the risk to suffer from tendon injuries during pregnancy. However, the variability in tendon stiffness values increased throughout pregnancy being 13% larger postpartum compared to early pregnancy. This indicates a highly individual response to the changed hormonal levels during pregnancy. Factors affecting the endocrine system in the postpartum phase, such as giving birth by cesarean section or breastfeeding (Atkinson and Leathem, 1946; Nissen et al., 1996), may have contributed to increased or reduced tendon stiffness in individual cases as well.

While in our study the mechanical properties of the patellar tendon did not change in general, pregnancy was found to affect its morphological properties. We detected a continuous increase in the tendon rest length measured at a 90° resting knee joint angle from early pregnancy to the postpartum measurement, which is also likely to be represented as a slight increase in the toe limit elongation. The implications of this change on the injury risk remain unclear. However, an increase in the tendon rest length may contribute to the increased joint laxity, which has frequently been reported for several peripheral joints in pregnant women. For the knee joint an increased amount of anterior tibial translation relative to the femur has been observed by Schauberger et al. (1996) using a clinical KT1000 arthrometer. Hypermobility measurements with gonio- and hyperextensometers in pregnant women demonstrated an increased range of motion in the elbow, the metacarpophalangeal joint of the index finger (Calguneri et al., 1982; Schauberger et al., 1996), the fourth finger (Östgaard et al., 1993), and the wrist (Marnach et al., 2003). A pregnancy induced increase in ligament rest length could explain why Hart et al. (2000) found an increase in knee joint laxity in pregnant rabbits while the stiffness of the medial collateral ligament did not change. Joint laxity during pregnancy is assumed to lead to instability in the joints (Ritchie, 2003), which may be associated with impairments in postural stability and an increased incidence of falls (Dunning et al., 2003; McCrory et al., 2010; Inanir et al., 2014). Thus, we cannot exclude that although tendon stiffness remained unchanged, the detected increase in tendon rest length may lead to an increased knee joint laxity. We may speculate that the increased rest length could result from effects due to hormonal changes and weight gain during pregnancy. It has been shown *in vitro* that increased levels of the hormone relaxin, which is elevated during pregnancy, potentiate creep effects in isolated rat tail tendons (Wood et al., 2003). An enhanced susceptibility to creep effects in combination with increased tendon load resulting from permanent weight gain during pregnancy may become apparent as long-term change in tendon morphology.

Apart from tendon properties we did not observe any effect of pregnancy on the knee extensor muscle strength. This is contrary to Atay and Basalan Iz (2015) reporting a 9% reduced handgrip strength at the end of pregnancy compared to the values being measured during the middle of pregnancy. The deviating findings may indicate that the upper and lower extremities undergo different adaptation processes during pregnancy. While loss in handgrip strength has been suggested to be primarily related to a reduced physical activity level (Atay and Basalan Iz, 2015) we may argue that leg strength has been maintained due to the pregnancy induced increase in body mass.

Thirty-two weeks after delivery the participants’ strength levels were similar to non-pregnant controls. This result was expected since muscle strength in the late postpartum phase has been reported to be almost consistent to the prior pregnancy status (Treuth et al., 2005).

It remains debatable whether the postpartum status actually reflects the prior pregnancy status. Fortunately, we were able to obtain data prior pregnancy in two women, allowing us to truly follow the development from prior pregnancy to the postpartum period in these two data sets. Confirming our conclusions, both women did not show a reduction in tendon stiffness with pregnancy while demonstrating an increase in tendon rest length. With a 16% increase in tendon rest length Woman-A appeared to be more sensitive to pregnancy-related changes while the increase in Woman-B was less with 6%, emphasizing the individual response to hormonal changes.

A limitation of our study is that our experimental setup did not consider measures of the length-tension properties of the tendon. As criticized by Hoang et al. (2007) this challenges the assessment of the true rest length. However, since it was not the purpose of this study to investigate changes in tendon rest length, but to determine changes in tendon stiffness, a potential pretension at 90° knee joint angle was not taken into account. For a better understanding of the tendon properties during pregnancy, future studies should include measurements of tendon rest length changes in a passive condition as well as in more than one loading condition using different knee flexion positions. In addition, it may aid understanding to assess further parameters such as muscle stiffness in combination with tendon and ligament properties.

Another limitation is that our results are mostly applicable to women having their first child as 15 of our 19 pregnant participants have delivered for the first time. Since research in rats has shown effects of repeated pregnancies on tendon properties (Rundgren, 1974) a higher percentage of women having their second or third child may affect the results. Furthermore, we would like to point out that we focused on a single tendon of the human body. It remains to be established if our findings are transferable to other types of tendons, since hormonal changes during pregnancy have been found to be tendon specific (Rundgren, 1974).

In conclusion, while the compliance of some ligaments such as the pelvic ligaments might change during pregnancy, we found no evidence to support the general assumption that tendons are subjected to the same change. Instead, our data provide evidence that patellar tendon stiffness is not affected by pregnancy. However, the progressive increase in the tendon rest length during and after pregnancy and its implication on the injury risk need to be further examined. Future studies are necessary to assess whether hypermobility in pregnant women may be related to a change in tendon or ligament rest length.

## Data Availability

The data sets generated for this study can be found in the Dryad Digital Respository: https://doi.org/10.5061/dryad.5s0860n.

## Author Contributions

KL conceived and designed the study. MEB and RM performed the data collection and further developed the advanced data analysis routines. MEB performed the data analysis and statistics, prepared figures, and wrote the first draft of this manuscript. LH assisted with the ethics application and was responsible for potential medical support of the subjects. KL and AA supervised the preparation of the manuscript and contributed to interpretation of the results. All authors approved the final manuscript and confirmed the responsibility of the content of this article.

## Conflict of Interest Statement

The authors declare that the research was conducted in the absence of any commercial or financial relationships that could be construed as a potential conflict of interest.
